# Curiosity for information predicts wellbeing mediated by loneliness during COVID-19 pandemic

**DOI:** 10.1038/s41598-022-11924-z

**Published:** 2022-05-11

**Authors:** A. B. Losecaat Vermeer, A. Muth, D. Terenzi, S. Q. Park

**Affiliations:** 1 Department of Decision Neuroscience and Nutrition, German Institute of Human Nutrition Potsdam-Rehbrücke, Nuthetal, Germany; 2 Charité-Universitätsmedizin Berlin, Corporate Member of Freie Universität Berlin, Humboldt-Universität Zu Berlin, Berlin, Germany; 3 Deutsches Zentrum Für Diabetes, Neuherberg, Germany

**Keywords:** Human behaviour, Nutrition, Social behaviour, Stress and resilience, Emotion

## Abstract

The COVID-19 pandemic confronted humans with high uncertainty and lockdowns, which severely disrupted people’s daily social and health lifestyles, enhanced loneliness, and reduced well-being. Curiosity and information-seeking are central to behavior, fostering well-being and adaptation in changing environments. They may be particularly important to maintain well-being during the pandemic. Here, we investigated which motives drive information-seeking, and whether and how curiosity and information-seeking related to well-being and mood (excitement, anxiety). Additionally, we tested whether daily diet contributed to this relationship during lockdown. Participants (*N* = 183) completed questionnaires measuring curiosity, information-seeking, social and mental health. Using a smartphone app, participants submitted their daily food intake and lifestyle ratings for a week. We found participants had highest motivation to seek positive (vs. negative) information, concerning themselves more than others. Both trait curiosity and information-seeking predicted higher well-being, mediated by loneliness. Trait curiosity also predicted well-being and excitement days later. Considering diet, participants with lower trait curiosity ate food containing more tyrosine (i.e., dopamine precursor). Furthermore, participants consuming food high in sugar reported higher anxiety, which was specifically found in participants with relatively low, but not high, trait curiosity. Taken together, curiosity and information-seeking may benefit well-being and mood in high uncertain and challenging times, by interacting with lifestyle measures (loneliness and nutrition).

## Introduction

The 2020 COVID-19 pandemic has confronted humans with high uncertainty and severely disrupted human societal daily life. Strict lockdowns induced a dramatic decrease in individual well-being, lifestyle changes including diet, and significantly reduced social interactions resulting in increased loneliness and stress^1,2^. Alongside these effects and the acute threat of the virus, the constant and unlimited information shared through news outlets and social media exposed individuals to vast amounts of information—accurate and inaccurate—not only regarding the coronavirus but also restrictions to daily lives^3^. Although information can aid decisions to obtain reward and avoid harm, individuals are generally motivated to avoid news they expect to have a negative impact on their feelings^4,5^. The abundance of COVID-19-related information likely enhanced uncertainty and feelings of anxiety, negatively impacting well-being^3^. Yet, how much knowledge is wanted in times of high uncertainty and loneliness, and how it relates to one’s well-being is unclear. A better understanding of these relationships during the pandemic is crucial for developing appropriate policies to provide adequate information and protect individuals from loneliness and poor mental health in the future.

Trait curiosity is commonly defined as the intrinsic desire for knowledge and seeking out novel, challenging situations^6^. This feeling, often compared to the feeling of hunger or thirst, is expressed in information-seeking behaviors such as reading, asking questions, conducting searches online, and watching the news. Both trait curiosity and its behavioral expression are fundamental for motivation, learning, and achieving and maintaining well-being^7–9^. For instance, trait curiosity is positively associated with life satisfaction and well-being^10^ and better coping with unfamiliar, uncertain information and challenging interactions^8^. Curiosity may facilitate such behaviors and promote well-being through positive emotions and reducing uncertainty. According to the broaden-and-build framework^11^, positive emotions including curiosity, broaden people’s scope of action and thinking, promote exploration and engagement with the environment to build knowledge and personal resources, and consequently regulate negative emotions and enhance psychological resilience. Hence, being curious and gathering information can be beneficial for coping with challenging situations. Moreover, what information people want to know (e.g., positive or negative) may reflect their mental state and mood^4^. Despite curiosity and information-seeking being integral to behavior, we know little about how it relates to well-being in an extensive period of social restrictions during an acute global health crisis.

Social interaction is critical for mental well-being^12^. In novel situations, we may be more eager to seek information about others (e.g., through observation) than by ourselves to minimize harm and avoid risk^13,14^, possibly demonstrating a self-other distinction in information-seeking. The imposed social restrictions reportedly increased loneliness by 20–30% during the pandemic, being highest during the first European lockdown in Spring 2020. Even later into the COVID-19 restrictions (i.e., late November 2020), 24% of 4436 adults partaking in a UK survey of the Mental Health Foundation (www.mentalhealth.org.uk) reported feeling lonely. Loneliness refers to the perceived social isolation and differs from objective social isolation (i.e., number of social interactions)^15^. Loneliness predicts negative life outcomes related to physical and mental well-being, including depression and anxiety^15,16^. However, not all individuals in this survey had been experiencing loneliness. Interestingly, loneliness has been associated with stable personality traits such as extraversion and openness^17,18^. Yet, it is unknown whether such a relation exists for trait curiosity. Consistent with the broaden-and-build framework, perceived loneliness can be lowered through positive emotions by building personal resources such as social support^19^. This may suggest being curious and motivated to seek information can build resources and reduce individuals’ loneliness, resulting in higher well-being. Hence, an open question is whether loneliness plays a mediating role in the relation between curiosity and mental well-being.

Besides social impact, lockdowns also affect our daily lifestyle^1^. Daily lifestyles including diet are hypothesized to significantly impact mental well-being. In particular, fruits and vegetables, and large amino acids including tyrosine and tryptophan predict outcomes such as positive mood, better mental health, and higher curiosity^20^, for a review^21,22^, whereas sugar intake had mixed effects, by predicting lower well-being but higher curiosity^20^. Tyrosine and tryptophan are essential precursors of neurotransmitters dopamine and serotonin, important for motivation and mood, respectively^23,24^. For instance, acute tyrosine and tryptophan depletions cause impairments in reward processing (e.g., reduced motivation for rewards) and lower mood^25,26^. Furthermore, studies have implicated a key role for dopamine in motivating information-seeking and exploration, as shown by the dopamine reward circuitry signaling the value of information^4,27,28^, and the relief of curiosity^29^. Based on these findings, changes in tyrosine and tryptophan from the food we eat may influence curiosity and information-seeking motivation, and thereby influencing our affective mental state. Simultaneously, lockdown-induced stress and loneliness may alter diet by preferring high-fat and high-carb food, as suggested by stress studies in animals^30^ and humans^31^, and thus negatively impact curiosity and well-being. A question we aimed to explore is whether there is any link between curiosity, both trait and behavior, with daily intake of macronutrients and amino acids during the lockdown, to predict well-being and mood.

**Figure 1:**
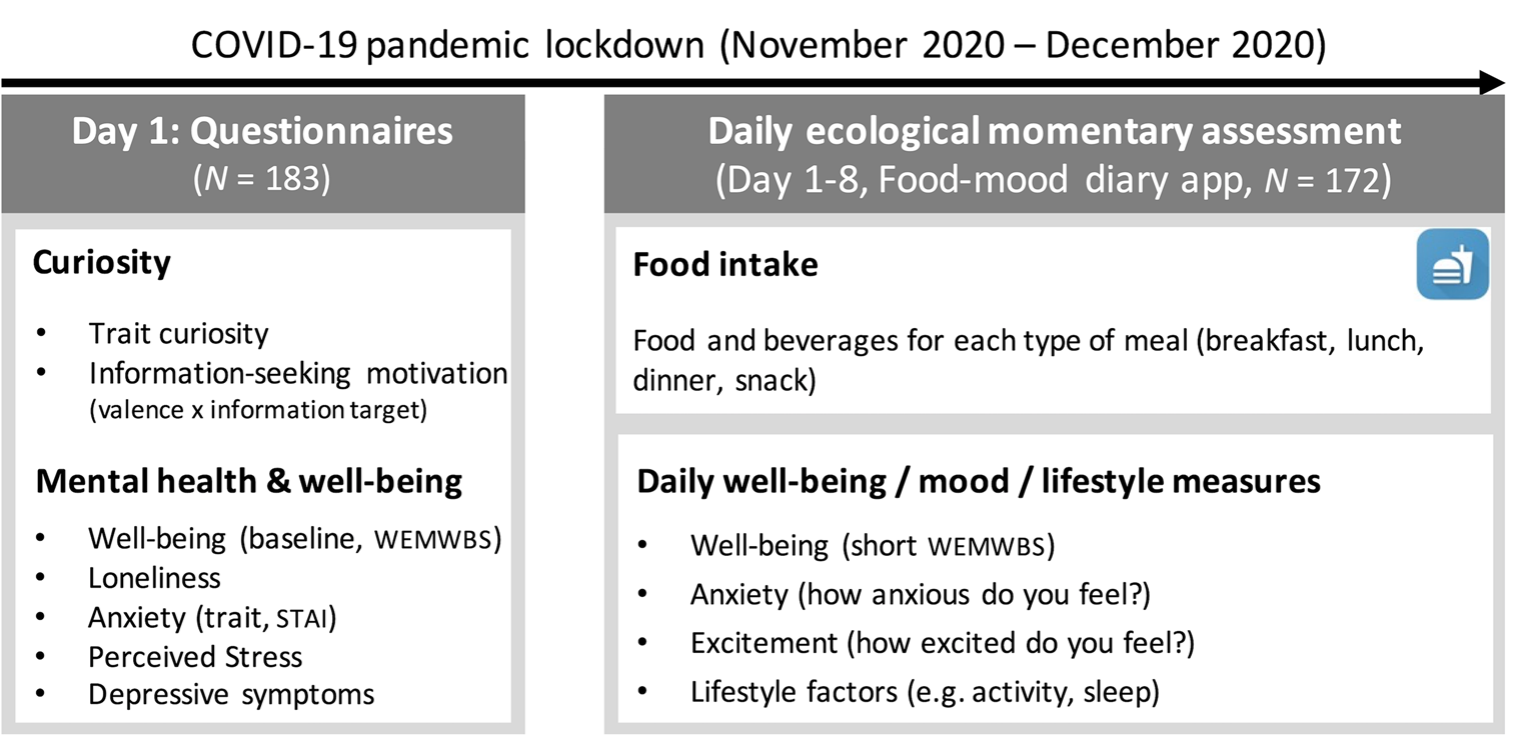
Outline of the study. On the first testing day, participants completed personality questionnaires measuring curiosity, mental well-being, demographics among others (see Methods). Subsequently, using the food-mood diary app, participants submitted their daily food intake for a week, and their ratings on well-being, mood, and other lifestyle factors once a day. *WEMWBS* Warwick Edinburgh Mental Wellbeing Scale, *STAI* State-Trait Anxiety Inventory.

In this preregistered study, we first investigated whether individuals are motivated to seek particular information specific for themselves and about others in an uncertain time involving increased loneliness, namely during the pandemic lockdown. Second, we examined how individual’s curiosity relates to mental well-being and mood (i.e. excitement and anxiety). We assessed both individuals’ trait curiosity level and their information-seeking motivation as an expression of curiosity. We hypothesized that individuals’ information-seeking motivation is dependent on the valence (i.e., higher for positive and lower for negative information) and the target of the information (i.e., information for oneself compared to information about others). Second, we hypothesized that curiosity (trait and behavior) is positively associated with individual’s mental well-being and mood (i.e., positive correlation with excitement and negative with anxiety). Third, we explored if relationships between curiosity with well-being and with mood are dependent on dietary intake during lockdown. To test these hypotheses, we conducted an online study in adults living in Germany and Austria (*N* = 183) during a national lockdown in the period from 10 November to 23 December 2020. Participants completed questionnaires assessing trait curiosity, information-seeking, loneliness, mental health, and COVID-19-related impact (e.g., work situation, regulation compliance) (see Fig. 1). Afterwards, participants submitted their self-rated levels of mood, well-being, and food intake for 7 days on average during lockdown.

## Results

Hundred eighty-seven participants signed up via Prolific for one of two preregistered online studies (i.e., one for Germany and one for Austria, see Methods). We excluded four participants with self-reported severe depression symptoms (i.e., Beck Depression Inventory score > 30, see Methods), resulting in 183 participants who completed the questionnaire battery. Of those participants, a total of 172 participants submitted their daily food intake and mood ratings for at least two days using the food-diary app. Therefore, analysis of daily mood and food observations were performed on these 172 participants. In Table 1, we provide descriptive statistics.

**Table 1:** Descriptive statistics.

Characteristic	Total sample (N = 183)^1^	Sample with diary data (n = 172)^1^*
Age	28.51 (8.94)	28.52 (8.98)
BMI	23.91 (4.44)	23.93 (4.06)
**Gender**		
Female	74/183 (40%)	69/172 (40%)
Male	109/183 (60%)	103/172 (60%)
**Nationality**		
Austrian	37/183 (20%)	34/172 (20%)
German	130/183 (71%)	128/172 (74%)
Other	16/183 (8.7%)	10/172 (5.8%)
**Country of residence**		
Austria	54/183 (30%)	46/172 (27%)
Germany	129/183 (70%)	126/172 (73%)
COVID-19 rules compliance (0–100)	82.33 (19.71)	81.70 (19.97)
Total diary submissions*		7.20 (1.13) (range = 2–8)

^1^Mean (SD); n/N (%).

*Minimum of 2 diary submissions.

### Drivers of information-seeking

Curiosity and information-seeking behavior can be driven by distinct motives. Generally, people are motivated to seek information they expect to have a positive impact on their feelings (valence-dependent), and avoid information that may have a negative impact on their feelings or mood^4,5^. In addition, people do not only acquire information on their own experiences, but also information directed towards and about others that can also be relevant for themselves. These types of information may separately or (most likely) concomitantly influence individual decisions and behavior^32^.

We first examined how individuals’ information-seeking behavior is driven by the valence (i.e., expected positive or negative information) and target of information (i.e., information concerning ourselves or about others). To test this, we asked participants how motivated they were at present to actively seek information (e.g., through searching the web, reading, asking questions) that was; (1) expected to be positive or negative, and (2) for themselves or about others. Participants rated their motivation on a slider from 0 to 100 (with 0 not motivated at all, 100 being highly motivated). This provided four ratings that allowed us to test potential drivers predicting information-seeking motivation, by performing a mixed-effects model. This model contained within-subject predictors for the valence (positive, negative) and the information target (self, other) and their interaction. Furthermore, we included random slopes (i.e., for valence and information target) and participants as random intercept.

**Figure 2:**
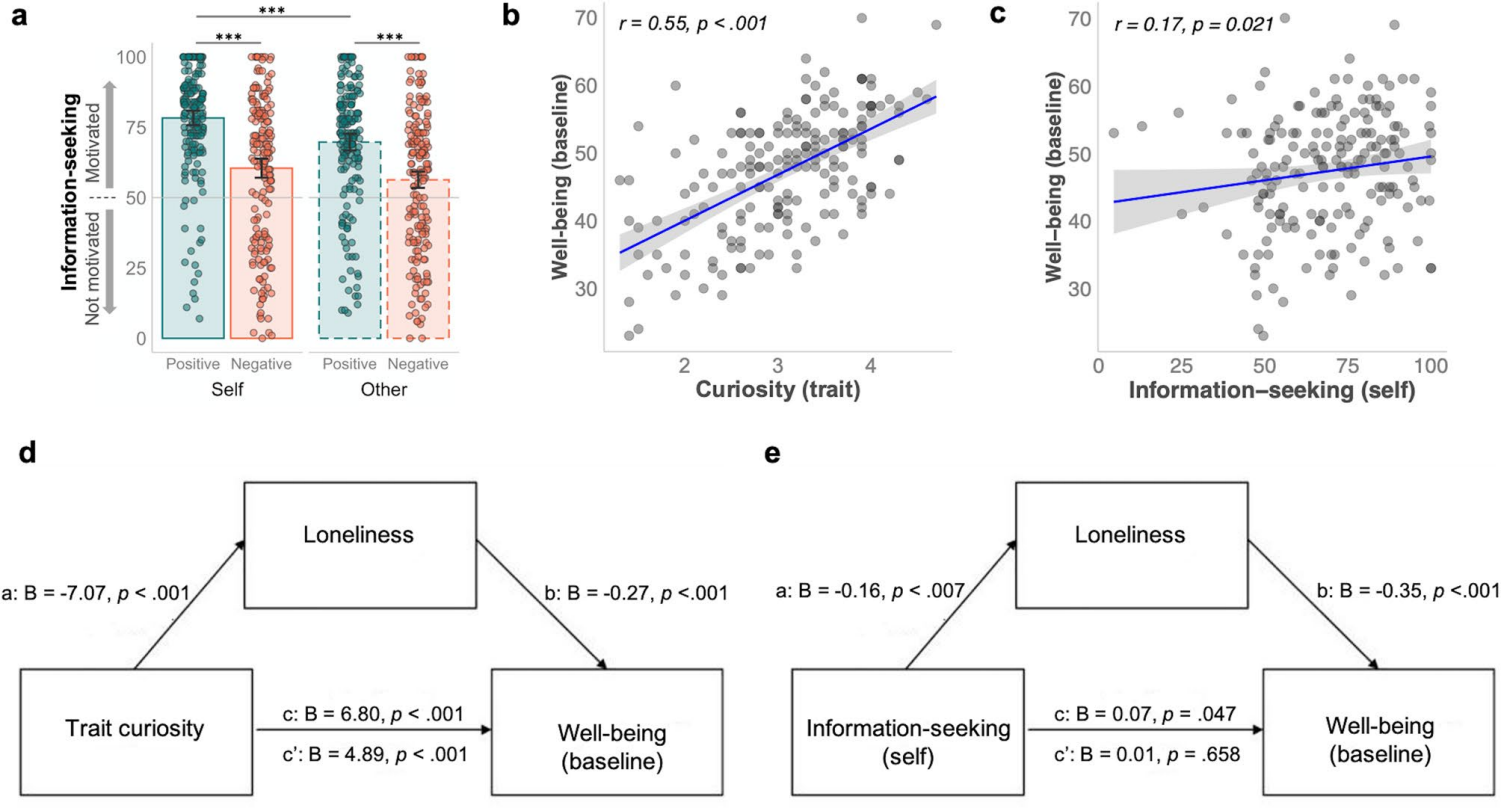
Information-seeking, curiosity, individual well-being (WEMWBS), and contributions of loneliness. (**a**) Information-seeking motivation is influenced by valence (positive, negative) and information target (self, other). Dots represent individual ratings. Error bars are SEM. Asterisks (*) indicate statistically significant differences: *** *p* < .001. (**b**) Correlation between trait curiosity and well-being. (**c**) Correlation between information-seeking for self on average and well-being. (**d**) Mediation model illustrating loneliness partially mediates the trait curiosity—well-being relationship. (**e**) Mediation model illustrating loneliness fully mediates the information-seeking for self—well-being relationship. Betas are unstandardized, total effect (c), direct path (c’).

Overall, participants were motivated to seek information (*M* = 66.22, *p* < 0.001, against 50 at which participants are neither motivated nor demotivated). Moreover, participants were more motivated to seek positive rather than negative information (i.e., valence-dependent, β = 7.81, *t*(182) = 8.16, *p* < 0.001), and information for oneself rather than about others (i.e., information target-dependent, β = 3.17, *t*(182) = 4.49, *p* < 0.001). Importantly, there was a significant valence by information-target interaction (β =  1.11, *t*(182) =  2.10, *p* = 0.037, Fig. 2a, Table S1), demonstrating that valence-dependent information-seeking motivation (i.e., positive–negative) was larger for self, compared to others (log_e_(*V*_Wilcoxon_) = 9.10, 95% CI [0.05, 0.38], *p* = 0.013, Fig. S1). This difference in motivation was mainly driven by seeking out potential positive information (self-other *M* = 8.56, *SE* = 1.77, *t*(377) = 4.85, *p* < 0.001; negative information: self-other *M* = 4.14, *SE* = 1.77, *t*(377) = 2.34, *p* = 0.091). In sum, individuals’ rated highest motivation to seek positive information concerning themselves.

### Trait curiosity and information-seeking

Trait curiosity was assessed via the Curiosity and Exploration Inventory-II scale (^8^, see Methods). As expected^8^, both subscales, stretching and embracing, correlated (r = 0.67, 95% CI [0.58, 0.75], *p* < 0.001). For this reason, the mean value of all items was taken as a measure of trait curiosity (range = 1.3–4.7, *M* = 3.08), with higher values indicating a relatively higher curiosity.

Curiosity typically manifests in information-seeking motivation and -behaviors. Indeed, correlating trait curiosity with the ratings of information-seeking, we found that participants with higher trait curiosity were more motivated to seek information for self on average (r = 0.25, 95% CI [0.10, 0.38], *p* = 0.004). In contrast, trait curiosity showed no significant relation with information-seeking in other conditions (i.e., about others: r = 0.13, 95% CI [− 0.02, 0.27], *p* = 0.306); with valence-dependent information-seeking (positive vs. negative): r =  − 0.05, 95% CI [− 0.20, 0.10], *p* = 0.476; or with information target-dependent information-seeking (i.e., self vs. other): r = 0.11, 95% CI [− 0.04, 0.25], *p* = 0.306). All p-values are Holm-corrected. These results demonstrate that trait curiosity relates to information-seeking motivation for self on average.

### Curiosity and information-seeking relate to mental well-being

One of the main aims was to investigate whether and how individuals’ curiosity and information-seeking relate to mental well-being during lockdown. Furthermore, we investigated if such potential associations are mediated by loneliness during COVID-19 restrictions.

Firstly, by computing correlations, we found that curious individuals who are, on average, more motivated to seek information for themselves also reported higher mental well-being at baseline (Warwick Edinburgh Mental Wellbeing Scale (WEMWBS), Fig. 2b,c). Secondly, participants with increased loneliness feelings reported significantly lower mental well-being (r =  − 0.60, 95% CI [− 0.69, -0.50], *p* < 0.001), had lower levels of curiosity (r =  − 0.35, 95% CI [− 0.47, − 0.21], *p* < 0.001), and were less motivated to seek information for oneself on average (r =  − 0.20, 95% CI [− 0.34, − 0.05], *p* = 0.014), *p*-values are Holm-corrected. These results support a relationship between curiosity measures, well-being, and loneliness.

Additionally, based on the valence- and information target-dependent effects on information-seeking, we tested whether these different motives of information-seeking correlated with individuals’ well-being. There was no significant correlation with valence-dependent information-seeking (positive–negative, r =  − 0.07, 95% CI [− 0.22, 0.08], *p* = 0.679), nor with the difference in information-seeking by target (self-other, r = 0.12, 95% CI [− 0.03, 0.26], *p* = 0.328), or solely information-seeking about others (r = 0.07, 95% CI [− 0.08, 0.22], *p* = 0.679). In summary, curiosity and well-being were associated with information-seeking for self on average, but not with information-seeking in the other conditions. Therefore, we continued subsequent analysis examining the link between information-seeking and well-being including lifestyle measures (i.e., loneliness, daily measures) on the average information-seeking ratings for self, as this measure demonstrated to relate to trait curiosity and well-being.

### Positive links between curiosity and well-being are mediated by subjective loneliness

We had preregistered to compute a multiple linear regression to assess the hypothesis that curiosity and information-seeking motivation relate to mental well-being, including loneliness as a covariate (Supplementary Information (SI)). However, as loneliness is integral to the pandemic lockdown and significantly correlates with both measures of interest, we opted for an alternative analysis approach. Specifically, to better understand how curiosity and information-seeking relate to mental well-being in a period with social restrictions, we examined whether loneliness mediated this relationship.

We computed one mediation model for trait curiosity as predictor of well-being with loneliness as mediator, and another mediation model for information-seeking as predictor (see Methods). Indeed, both models revealed loneliness as a mediator. The relationship between trait curiosity and well-being was partially mediated by loneliness, as the direct effect reduced in size (c’: B = 4.89, *SE* = 0.63) compared to the total effect (c: B = 6.80, *SE* = 0.71, Fig. 2d; bootstrapped indirect effect (a*b) B = 1.92, *SE* = 0.44, Z = 4.35, *p* < 0.001, R2 = 0.53). Interestingly, loneliness fully mediated the effect of information-seeking on well-being (R2 = 0.38, bootstrapped indirect effect (a*b) B = 0.08, *SE* = 0.02, Z = 2.60, *p* = 0.009, Fig. 2e), such that information-seeking predicted mental well-being, by influencing individuals’ feelings of loneliness.

Taken together, we found that the subjective loneliness during the COVID-19 pandemic fully mediates the positive relationship between information-seeking and mental well-being, and partially between individuals’ trait curiosity and well-being. Interestingly, trait curiosity also still had an incremental direct effect on well-being independent of subjective loneliness.

### Daily well-being and excitement during lockdown are linked to individuals’ trait curiosity

So far, we showed that both trait curiosity and information-seeking behavior are associated with baseline mental well-being (WEMWBS) measured at day one. Next, we also aimed to investigate whether these curiosity measures assessed at day one could predict individuals’ averaged daily well-being (i.e., short WEMWBS) and mood (i.e., excitement and anxiety) reported during the rest of the lockdown week, hence having a longer-lasting predictive effect.

### Trait curiosity, daily well-being, and mood

**Figure 3:**
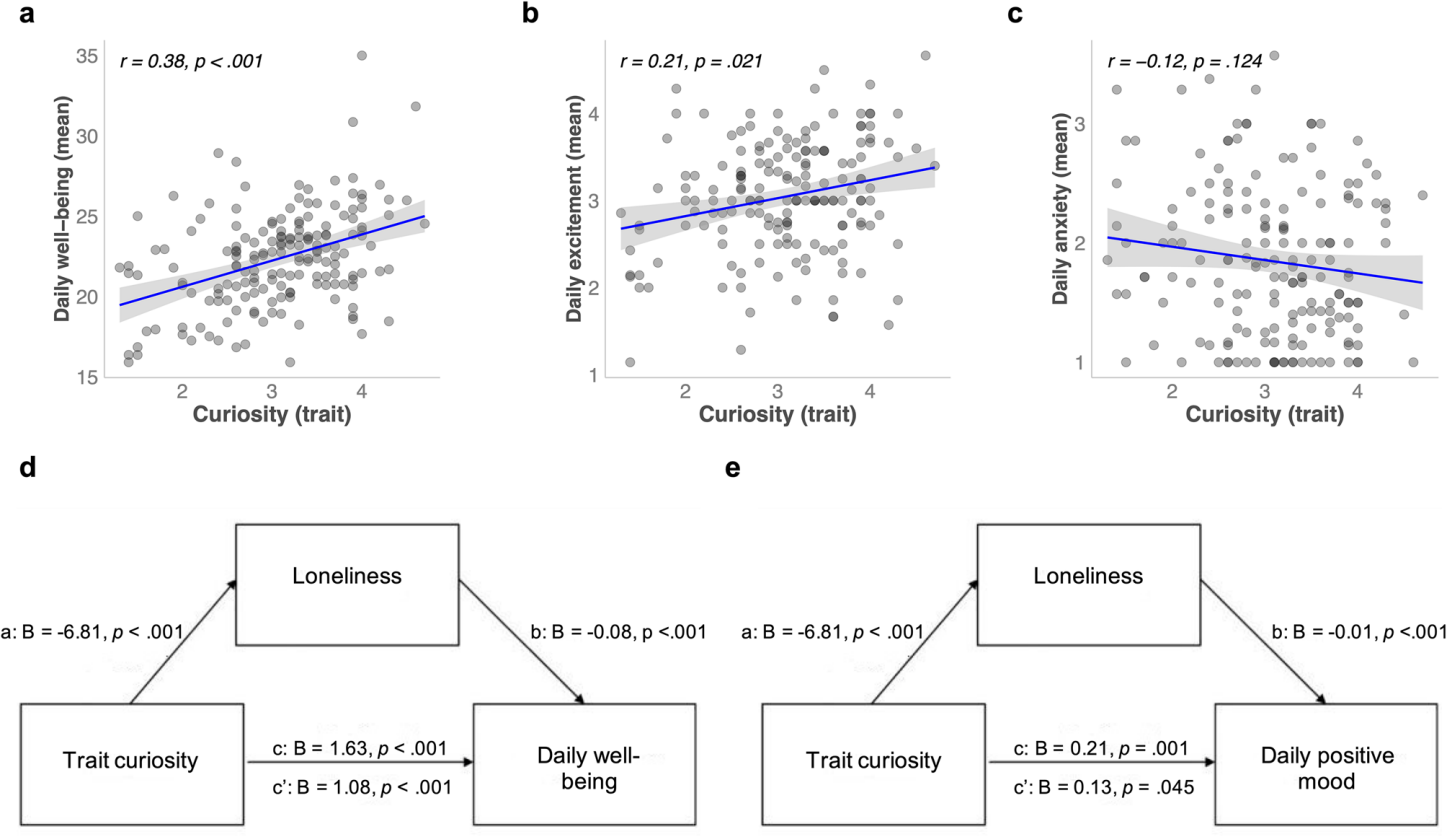
Associations between trait curiosity, daily well-being, and mood. Significant correlations between trait curiosity, daily well-being (**a**), and daily excitement (**b**), but not with daily anxiety (**c**). Mediation models with trait curiosity as predictor and loneliness as mediator predicting daily well-being (**d**), and daily excitement (positive mood) (**e**). Betas are unstandardized, total effect (c), direct path (c’).

We found a positive correlation between trait curiosity with individuals’ average daily well-being (Fig. 3a), and excitement (Fig. 3b), but not with anxiety (Fig. 3c). These associations were also observed when including trait curiosity and loneliness as predictors and covariates for age, gender, and residency (and trait anxiety level, for anxiety model, see Methods) into a multiple regression (well-being: β = 1.06, 95% CI [0.50, 1.63], *p* < 0.001; excitement: β = 0.16, 95% CI [0.03, 0.29], *p* = 0.019; anxiety: β = 0.08, 95% CI [− 0.05, 0.21], *p* = 0.237).

Next, we investigated whether loneliness also mediated the relationship between curiosity and daily well-being and excitement. The mediation models revealed a significant indirect effect predicting average daily well-being (bootstrapped indirect effect (a*b): B = 0.55, *SE* = 0.15, Z = 3.68, *p* < 0.001, R2 = 0.31), and predicting average daily excitement (bootstrapped indirect effect (a*b): B = 0.07, *SE* = 0.03, Z = 2.74, *p* = 0.006, R2 = 0.12), illustrating that loneliness partially mediated the relationship between trait curiosity and daily well-being and excitement (Fig. 3d,e).

### Information-seeking does not predict daily well-being and mood

Information-seeking overall for self did not significantly predict averaged daily well-being (*F*(1, 170) = 1.43, *p* = 0.234), daily excitement (*F*(1, 170) = 2.31, *p* = 0.130), or daily anxiety (*F*(1, 170) = 0.28, *p* = 0.598), nor when including covariates (SI). In sum, the association between information-seeking and individuals’ baseline well-being (WEMWBS) was not observed for average well-being, days later into lockdown.

### Curiosity, daily dietary intake, and their influence on daily mood

We preregistered to explore whether there is a link between an individual’s average daily food intake (i.e., nutritive content) and curiosity to predict daily well-being and mood. Moreover, based on associations between dopamine and curiosity and information-seeking^4,5,27,29^, we explored whether trait curiosity and information-seeking could predict the daily intake days later of large neutral amino acids (LNAA) tyrosine- and phenylalanine (i.e., precursor for dopamine), and tryptophan (precursor for serotonin) relative to other LNAAs^33,34^. We extracted the intake of macro-and micro-nutrients including LNAAs from the submitted food in the food-diary app (see Methods).

First, exploring whether trait curiosity and information-seeking associated with intake of tyrosine/LNAA including tyrosine- and phenylalanine/LNAA, and tryptophan/LNAA, we ran a linear regression including gender, because of observed gender differences in food intake (see Table S4). Trait curiosity negatively predicted tyrosine- and phenylalanine/LNAA intake (β =  − 0.003, *p* = 0.017), such that individuals with lower trait curiosity consumed more tyrosine-rich food. Information-seeking motivation also negatively predicted the intake of tyrosine-rich food (i.e., tyrosine/LNAA, β =  − 0.000, *p* = 0.014). Tryptophan/LNAA ratio was neither predicted by trait curiosity (*p* = 0.151), nor information-seeking motivation (*p* = 0.964). Although the interaction between trait curiosity and gender for tryptophan/LNAA intake was statistically non-significant (*p* = 0.064), the data tends to indicate that women with lower trait curiosity consume more tryptophan-rich food (β = − 0.001, *p* = 0.015) compared to men (β = 0.000, *p* = 0.777).

**Figure 4:**
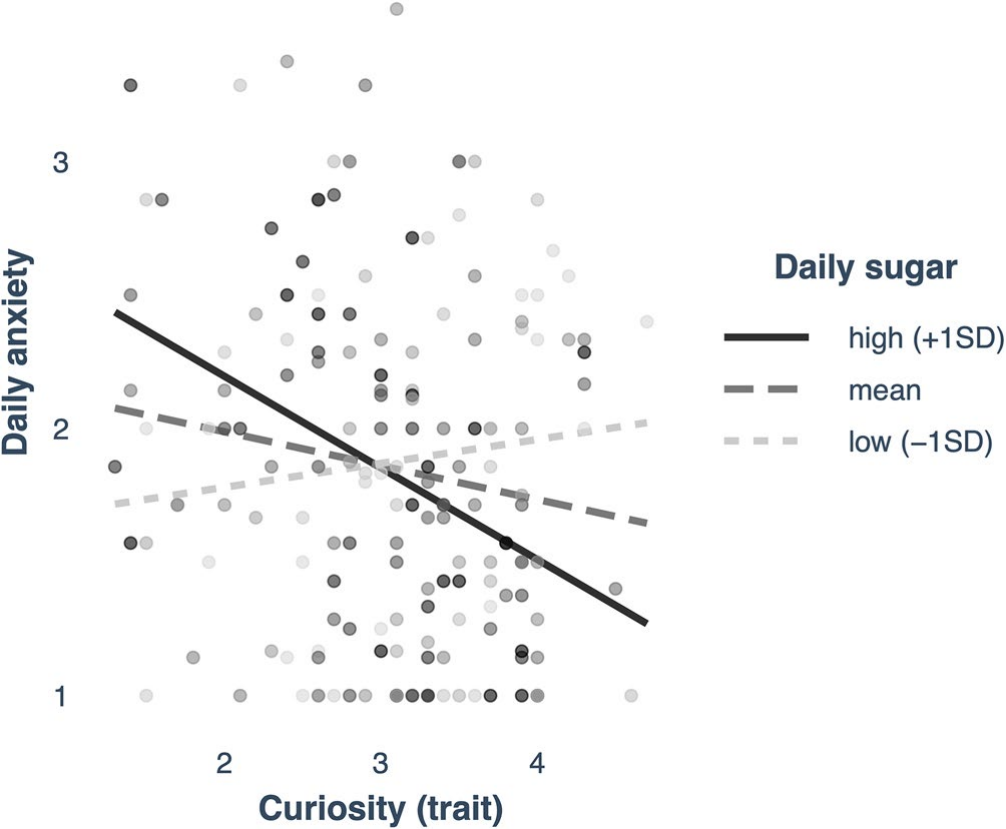
Relationship between trait curiosity and daily anxiety at different levels of sugar intake (high, mean, low).

Next, we examined whether food intake indexed by certain nutrient compositions (i.e., carbs, fat, simple sugars, fruit and vegetables) contribute to the curiosity—daily well-being and mood link. As information-seeking did not show any association with self-rated daily well-being and mood, we focused on trait curiosity only. We included the nutrient compositions (as mean percentage) and trait curiosity into linear models including potential covariates (e.g., gender, residency, age) to predict daily well-being, excitement, as well as anxiety (i.e., three independent models). Food intake showed no significant main effects and interactions with trait curiosity predicting well-being or excitement (all *p*’s < 0.05, see Table S5 + S6). However, we found that sugar intake positively predicted anxiety (β = 0.25, *p* = 0.008), which was influenced by individuals’ trait curiosity (interaction: β = − 0.08, *p* = 0.005, Fig. 4, Table S7). Further decomposing this interaction, by comparing the slopes, showed that sugar intake increased anxiety in participants with low trait curiosity, which effect was reversed in participants with high trait curiosity (high sugar, β =  − 0.34, Z = − 3.64, *p* < 0.001). This was not observed for mean (β =  − 0.13, Z =  − 1.98, *p* = 0.053) to low sugar intake levels (β = 0.09, Z = 1.04, *p* = 0.308).

## Discussion

The COVID-19 pandemic has profoundly affected our daily social lives, physical and mental well-being^1,2^. Trait curiosity and access to adequate information are hypothesized to promote better coping with challenging situations, enhancing well-being, mood, and life satisfaction^6–9^. In the current study, we investigated three main questions. We asked which motives drive information-seeking, and whether trait curiosity and information-seeking motivation are linked to individuals’ mental well-being and mood during lockdown in a global health crisis, encompassed of extreme loneliness and uncertainty. Furthermore, we investigated whether the relationship between curiosity, well-being, and mood is moderated by the food we consume during the lockdown.

Our results show that both trait curiosity and information-seeking motivation for self on average predicted higher well-being and reduced loneliness during lockdown. In line with previous findings^15,16^, we also found a negative association between loneliness and lower well-being. Interestingly, we found that the association between curiosity and information-seeking with well-being was mediated by individuals’ feelings of loneliness. Thus, our data suggest that individuals’ information-seeking for self and curiosity is beneficial for well-being, through reducing loneliness during the lockdown. Moreover, trait curiosity also still predicted well-being independently.

Consistent with the broaden-and-build framework^11^, being curious and gathering information may foster positive states and coping with social restrictions by reducing loneliness feelings through increased engagement and exploration. Individual differences in loneliness and resilience have previously been linked with personality traits^17,18^, with people scoring higher on extraversion and openness reporting lower loneliness feelings and higher well-being. Our findings contribute to this, by showing that a person’s trait curiosity and momentary information-seeking motivation also predict higher well-being, mediated by a lower level of loneliness during a socially challenging and uncertain time.

Trait curiosity, but not information-seeking, also predicted daily well-being and excitement (i.e. positive mood) on a longer timescale—i.e., averaged across 2–8 observation days into the lockdown. In contrast with trait curiosity^8^, we explicitly asked participants about their motivation for seeking information at the present time, likely capturing their momentary state and motivation to interact with their environment. Hence, this measure may vary within participants across subsequent days, and consequently might not be a good predictor of daily well-being and mood at later timepoints. Indeed, recent work found that consistency in state curiosity is an important predictor of daily well-being^35^. These findings suggest that an individual’s trait-level curiosity and consistency in state-level curiosity is important to predict well-being and mood daily, while the momentary motivation to seek information as a behavioral expression of one’s curiosity likely relates to well-being and mood on a shorter timescale.

Replicating studies on information-seeking^28,36–39^, individuals' were more motivated to seek positive rather than negative information. Additionally, we found that this valence-dependent effect was stronger for own information than information about others, and driven mostly for information that was positive but not negative. While people are generally motivated to seek positive information because of the expected positive feelings from having a positive belief^28^, good news about others may provide less strong positive feelings. Likewise, vicarious rewards produce less strong reward-related activation than own rewards^40^. However, social information concerning negative outcomes can still be important in novel situations and when the quality of information is uncertain, to avoid being exposed to risks from first-hand experience.

When feeling lonely, individuals may even be more motivated to seek information about others^16,41^. Surprisingly, our results showed a non-significant negative relation between information-seeking about others and loneliness, suggesting that feeling lonely does not relate to the willingness to know more about others' outcomes. This is in slight contrast to other findings^16^, demonstrating that people with higher loneliness over-attend to negative social information in the environment. We also did not find a correlation between loneliness and the motivation to seek negative information about others. Instead of providing participants with information, we asked participants whether they wanted information about others. The difference between passive information and active information-seeking could explain why we did not find a relation between loneliness and information-seeking concerning others.

Despite showing valence-dependent motivation to seek information, the link between mental well-being and information-seeking motivation was independent of valence. Seeking information, both positive and negative, can help reduce uncertainty^5^. Moreover, previous findings reported that more curious individuals are better capable in regulating and controlling their emotions, and report higher well-being^8^. Hence, our data further suggest that higher well-being relates to high curiosity and motivation to explore knowledge involving both positive and negative valence information.

Besides the social context, the lockdown also resulted in lifestyle changes including diet^42^. Healthy eating can protect one’s mental health and mood, particularly in challenging and stressful times^20–22^, with stress predicting lower quality food intake full of carbs, sugars, and fat^30,31^. Nonetheless, in a previous study^20^, fruit and vegetable intake increased well-being and curiosity, although sugar intake decreased well-being but increased curiosity. We explored whether food intake contributed to the effects of curiosity on mood and well-being. In contrast to Conner and colleagues^20^, we did not observe a relation between fruit and vegetable intake and curiosity, nor did it predict mood or well-being. However, we found a significant interaction between sugar intake and trait curiosity predicting feeling more anxious during the lockdown. Food high in sugar predicted higher anxiety among people with low trait curiosity and lower anxiety among those with high trait curiosity. This suggests that high trait curiosity may buffer negative effects of high sugar on mood, whereas individuals low in trait curiosity—who typically explore less and regulate their emotions less well, may be particularly susceptible to the negative effects of high sugar^43^. Consistent with this, previous studies have linked high-sugar/fat intake to enhanced anxiety (for review^44^) and changes in dopamine precursor availability^45^. Possibly, curiosity compensates for the effects of sugar on anxiety through its effect on positive mood, motivation, and enhanced mesolimbic dopamine activity^8,28^. Compared to prior food diary studies e.g.,^20^ that assessed self-reported food intake on predefined categories (i.e., sweets, fruit, vegetables, and chips), our study takes a more holistic approach by extracting nutrients as dietary components from the quantity of each food and beverage participants consumed over the day for a week, using a food-diary app.

Protein-rich food contains high amounts of LNAAs including dopamine and serotonin precursors^33,46^. The reward circuitry, in particular dopamine, has been associated with curiosity and information-seeking^28,47^. We found that individuals with lower trait curiosity and information-seeking motivation consumed more tyrosine-rich food, providing an indirect link between dopamine and curiosity. Individuals low in curiosity may require foods high in tyrosine to increase dopamine synthesis to regulate their information-seeking motivation and mood. Supporting this, atypical information-seeking motivation has been linked to abnormal dopamine functioning. For instance, enhancing dopamine through levodopa was found to increase information gain for negative non-instrumental information, without affecting mood^48^. Moreover, there was no observed link between curiosity and food intake containing high-tryptophan levels, potentially suggesting that tryptophan plays a less prominent role in curiosity, or could have specific effects that our measures could not capture e.g.^49^. The causal effect of our findings remains open. Future studies should test for the direction of effects of diet and curiosity, for example, using a dietary intervention containing high/low precursors of dopamine and serotonin.

Some limitations and constraints should be considered. First, curiosity and loneliness were only measured once at the beginning of the study, therefore we cannot reveal within-person changes over time and test how they relate to daily changes in well-being and mood during lockdown. Second, despite the positive contributions of curiosity and food on well-being and mood, these findings are strictly correlational and we cannot draw a causal link. It is plausible that the relationships reported here are bi-directional (e.g., food predicts curiosity, as shown for behavior^33^). Intervention experiments are required to establish whether a bi-directional association exists. Third, daily food and mood measures were based on self-report, which suffers from reliability issues and accuracy. Despite this, a strength is that it provided a rich dataset with multiple daily momentary observations, representing a more ecological representation of people’s daily food intake under real-life conditions.

In conclusion, trait curiosity and information-seeking motivation predicted higher well-being, investigated during lockdown of COVID-19 pandemic. Importantly, loneliness played an underlying mediating role. Furthermore, high-sugar intake increased anxiety during lockdown, specifically in people with low trait curiosity. The causal and exact direction of effects reported here need to be corroborated by future research, and whether these effects can generalize to other socially challenging contexts. In the current study, information-seeking motivation was assessed for topics broadly related to the pandemic (e.g., COVID-19, finance, health). Our results suggest that information-seeking concerning—but not exclusive to the pandemic—can predict well-being in times of a pandemic, mediated by perceived loneliness. It would be important for future studies to investigate the change in information-seeking behavior in normal (pandemic naïve) compared to emergent (pandemic) times. Our results suggest that curiosity interacts with lifestyle measures (loneliness and diet), indicating the potential to modulate each of these players to potentially increase well-being and mood during a time of crisis.

## Methods

### Participants

Hundred eighty-three participants were accepted to partake in the online study in the period from 10 November to 23 December 2020 during light and strict lockdown phases in Germany and Austria, respectively. During these lockdown periods, people were asked to reduce social contacts to the necessary minimum and mainly work from home (see https://www.bundesregierung.de/breg-de/themen/coronavirus/corona-massnahmen-1734724; from: 28.10.2020; https://www.sozialministerium.at/Informationen-zum-Coronavirus/Coronavirus---Aktuelle-Maßnahmen.html, from 17.11.2020). Participants were recruited via the Prolific platform (https://prolific.co/). Inclusion criteria were living in Germany or Austria at the time of the study, fluent in German, and no history of psychiatric illness. For the second part of the online study, participants had to log their food intake and their mood daily via a smartphone app (i.e., FoodApp). A priori inclusion criteria were set to a minimum of four days of food and mood entries per person. However, as we preregistered to calculate the average mood and food intake, and to minimize loss of valuable data, we decided to restrict our sample to a minimum of two completed entries for food intake and mood ratings. This resulted in data of a total of 172 participants. Participants received £28 for their participation. This study was preregistered (https://osf.io/xfcwb; https://osf.io/tgpr4). The study procedure was performed in accordance with the Declaration of Helsinki and approved by the ethical committee board of the Humboldt University Berlin (code 2020–22).

### Measures

#### Information seeking motivation

We asked participants to rate their information-seeking motivation on four questions. Participants rated their motivation on a sliding bar from 0 to 100 (i.e., not motivated to seek information—highly motivated to seek information) for positive/negative expected outcomes related to oneself and about other people. A few identical topics—directly and indirectly related to the COVID-19 pandemic—were given as an example of positive and negative information (see SI for questions and exact description). The questions occurred in a fixed order, with questions referring about others on a separate page to minimize direct comparison. Participants did not know in advance they would also be asked to also rate their information-seeking motivation about others. These questions appeared in a battery of questionnaires assessing for instance personality traits, and COVID19-related impact, minimizing potential demand effects.

#### Trait curiosity

Individual’s trait curiosity was assessed via a validated questionnaire (Curiosity and Exploration Inventory-II (CEI-II); 10 items^8^). This scale assesses people’s disposition to explore unsolved problems, unpredictable challenges, and unfamiliar experiences, all out of a sense of curiosity about the world. Example items include, “I actively seek as much information as I can in new situations” and “I prefer jobs that are excitingly unpredictable.” Answers are given on a 5-point scale (1 = very slightly or not at all; 5 = extremely). The CEI-II had good internal reliability (Cronbach's α = 0.87, 95% CI [0.84, 0.90]).

### Mental well-being (baseline), loneliness, affective health, and COVID-19-related impact

Participants completed validated standardized self-report questionnaires assessing a range of mental and affective health symptoms. These included mental well-being (Warwick Edinburgh Mental Wellbeing Scale (WEMWBS), Cronbach's α = 0.91, 95% CI [0.88, 0.92])^50^, perceived loneliness (UCLA Loneliness Scale, Cronbach's α = 0.94, 95% CI [0.93, 0.95])^51^, depression (Beck’s Depression Inventory, Cronbach's α = 0.82, 95% CI [0.77, 0.85])^52^, trait anxiety (State-Trait Anxiety Inventory, Cronbach's α = 0.82, 95% CI [0.79, 0.85])^53^, perceived stress (Perceived Stress Questionnaires, Cronbach's α = 0.91, 95% CI [0.89, 0.93])^54^, and also other lifestyle-related questions that were part of a larger study (preregistered under https://osf.io/nqhjf) that were not analyzed here. Furthermore, we assessed the impact COVID-19 had on participants’ lives such as how well they complied with the rules, what their current work situation was (e.g., home-office), and whether they or others they knew contracted the virus (see SI for details).

### Daily food, well-being, and mood

Once a day after 17:00 h participants are asked questions about their mental well-being, mood (i.e. feeling excited, feeling anxious), and lifestyle (e.g., activity) via the food-diary app.

Daily well-being was assessed using the short WEMWBS. Participants were asked to rate how they felt on a scale from 1 to 5, on 7 items. Total scores ranged from 7 to 35, with higher scores reflecting higher levels of well-being. Mood was assessed for anxiety and excitement using the two items from the Positive Affect Negative Affect Scale^55^. Participants were asked to rate both items on a scale from 1 to 5. Furthermore, participants were also asked to rate how well they slept, how active they were that day, and about quantity and satisfaction of their social interactions that day on a visual analog scale from 1 to 100.

Food intake was assessed via self-report using the food-diary app. Here, participants provided the following information: date and time, type of meal (e.g., breakfast, lunch, snack), whether they ate alone or in company, the food item and quantity (in grams or milliliters). Participants are asked to complete the daily food diary and mood questions for 7 days. The output allowed us to extract caloric content and information on macro- and micronutrients of the consumed food using the German BLS data table^56^. We excluded observations from analysis that fell outside the plausible daily energy (caloric) intake, i.e., < 500 kcal/day and > 3500 kcal/day for women, and < 800 kcal/day or > 4000 kcal/day for men^57^, and winsorized remaining outlier values separately by gender^58^.

Food intake was adjusted for individual energy intake (g/1000 kcal/day) as suggested by^59^. We multiplied the daily intake of carbohydrates (g/day) by 4 kcal, fat intake by 9 kcal, sugars by 2 kcal to obtain the daily energy derived from each macronutrient (Table S4). Tyrosine and tryptophan to LNAA ratios were calculated by dividing the quantity of tyrosine and tryptophan by the sum of the other LNAAs (Eqs. 1a, 1b, 2), and used as a proxy of brain tyrosine and tryptophan levels and ultimately brain dopamine and serotonin levels, respectively^33^.

1a $${\text{Tyrosine}} + {\text{Phenylalanine ratio}} = \frac{{\left( {{\text{Tyrosine}} + {\text{Phenylalanine}}} \right){ }}}{{{\text{sum}}\left( {{\text{Tryptophan }} + {\text{ Leucine }} + {\text{ Isoleucine }} + {\text{ Valine}}} \right)}}$$

1b $${\text{Tyrosine ratio}} = \frac{{{\text{Tyrosine}}}}{{{\text{sum}}\left( {{\text{Tryptophan }} + {\text{ Phenylalanine }} + {\text{ Leucine }} + {\text{ Isoleucine }} + {\text{ Valine}}} \right)}}$$

2 $${\text{Tryptophan ratio}} = \frac{{\text{Tryptophan }}}{{{\text{sum}}\left( {{\text{Tyrosine }} + {\text{ Phenylalanine }} + {\text{ Leucine }} + {\text{ Isoleucine }} + {\text{ Valine}}} \right)}}$$

### Study design

After providing instructions and written informed consent, participants were invited to complete a battery of questionnaires online assessing trait curiosity, information-seeking motivation, mental health (e.g., mental well-being, loneliness, stress, anxiety), and lifestyle behaviors (including sleep, activity, and social factors).

Next, participants were asked to install a food-diary app provided by us on their smartphone and submit a daily diary of their food consumption (i.e., daily intake of food items and beverages) in the app, from which we extracted the total calories, macro- and micronutrients per meal per day. They were also asked to rate their well-being and mood (on a scale from 1 to 5) once a day for a week using the same app. Furthermore, daily subjective experiences regarding sleep quality, activity, and social interactions were also recorded (Fig. 1).

Due to the timing of the lockdown restrictions, we first conducted the online study in Germany as preregistered. While the German study was ongoing, the government in Austria announced an upcoming lockdown. Therefore, we decided to repeat the same experiment in Austria (separately preregistered with the same questions and measures). After data collection and because of the small sample in Austria, we instead merged the data of the German sample and the first Austrian sample and included a predictor of residence country in the analysis to account for potential differences in experienced lockdown measures. We chose Austria, as Austria and Germany are geologically close, share the same native language, and are highly comparable both on a social and economic level.

### Statistical analysis

The analysis plan was preregistered on the public data repository Open Science Framework (https://osf.io/xfcwb; https://osf.io/tgpr4). The data were analyzed using R statistical software (R Core Team, 2020).

First, we investigated which motives drive information-seeking motivation. To test this, we ran a mixed effect model on the ratings for information-seeking with within-subject predictors for the valence and information target, including by participant random intercept and random slopes for the within-subject predictors (i.e., valence and information target). Mental well-being was measured once at the beginning of the study (baseline, WEMWBS) and through the app as a day-to-day well-being measure (daily well-being, short WEMWBS). Daily measures of well-being, excitement and anxiety were each averaged per person over the days logged. To test the relationships between trait curiosity, information-seeking motivation, and the dependent variables mental well-being (baseline and daily), mood (excitement, anxiety), and food intake, we computed non-parametric (Spearman) correlations and linear regressions. To control for individual differences, we included covariates age, gender, and country of residence in the linear models. To avoid biased coefficients due to high correlation, we did not include baseline well-being scores as a covariate in the model to predict average daily well-being, and perceived stress (PSQ) in combination with trait anxiety to predict daily anxiety. Analysis was performed using “lme4” (version 1.1.27)^60^ and “lmerTest”(version 3.1.3)^61^. P-values for linear models were computed using Kenward-Roger approximation. Post-hoc analyses were done using “emmeans” (version 1.6.1)^62^, and “interactions” (version 1.1.0)^63^ for simple slope comparisons, and adjusted for multiple comparisons using Tukey where relevant. For correlations, p-values were adjusted using Holm correction (1979). Note in the preregistration we reported to use the Bonferroni as correction for multiple comparisons, however, we decided for Tukey and Holm instead as it is considered uniformly more powerful. Table and plots were created using “gtsummary” (version 1.4.1)^64^ and “ggplot2” (version 3.3.3)^65^. Mediation analyses were performed to test whether the relationship between information-seeking, trait curiosity, and the dependent variables was mediated by feelings of loneliness. Bootstrapping (1000 samples) was performed as implemented in the psych package (version 2.1.3)^66^.

## Supplementary Information

Supplementary Information.

## Data Availability

The anonymized datasets and code from the current study are available on the Open Science Framework (https://osf.io/z4qmu/).

## References

[1] Brooks SK (2020). The psychological impact of quarantine and how to reduce it: Rapid review of the evidence. Lancet.

[2] Gubler DA, Makowski LM, Troche SJ, Schlegel K (2020). Loneliness and Well-Being During the Covid-19 Pandemic: Associations with Personality and Emotion Regulation. J. Happiness Stud..

[3] Bendau A (2021). Associations between COVID-19 related media consumption and symptoms of anxiety, depression and COVID-19 related fear in the general population in Germany. Eur. Arch. Psychiatry Clin. Neurosci..

[4] Sharot T, Sunstein CR (2020). How people decide what they want to know. Nat. Hum. Behav..

[5] van Lieshout LL, de Lange FP, Cools R (2020). Why so curious? Quantifying mechanisms of information seeking. Curr. Opin. Behav. Sci..

[6] Loewenstein G (1994). The psychology of curiosity: A review and reinterpretation. Psychol. Bull..

[7] Kashdan TB, Steger MF (2007). Curiosity and pathways to well-being and meaning in life: Traits, states, and everyday behaviors. Motiv. Emot..

[8] Kashdan TB (2009). The curiosity and exploration inventory-II: Development, factor structure, and psychometrics. J. Res. Pers..

[9] Kidd C, Hayden BY (2015). The psychology and neuroscience of curiosity. Neuron.

[10] Peterson C, Ruch W, Beermann U, Park N, Seligman MEP (2007). Strengths of character, orientations to happiness, and life satisfaction. J. Posit. Psychol..

[11] Fredrickson BL (2004). The broaden–and–build theory of positive emotions. Philos. Trans. R. Soc. Lond. Ser. B Biol. Sci..

[12] Kawachi I, Berkman LF (2001). Social Ties and Mental Health. J. Urban Health Bull. N. Y. Acad. Med..

[13] Rendell L (2010). Why copy others? Insights from the social learning strategies tournament. Science.

[14] Boyd R, Richerson PJ, Henrich J (2011). The cultural niche: Why social learning is essential for human adaptation. Proc. Natl. Acad. Sci..

[15] Hawkley LC, Cacioppo JT (2010). Loneliness matters: A theoretical and empirical review of consequences and mechanisms. Ann. Behav. Med..

[16] Cacioppo JT, Hawkley LC (2009). Perceived social isolation and cognition. Trends Cogn. Sci..

[17] Buecker S, Maes M, Denissen JJA, Luhmann M (2020). Loneliness and the big five personality traits: A meta-analysis. Eur. J. Pers..

[18] Modersitzki N, Phan LV, Kuper N, Rauthmann JF (2020). Who is impacted? Personality predicts individual differences in psychological consequences of the COVID-19 pandemic in Germany. Soc. Psychol. Personal. Sci..

[19] Newall NEG, Chipperfield JG, Bailis DS, Stewart TL (2013). Consequences of loneliness on physical activity and mortality in older adults and the power of positive emotions. Heal. Psychol..

[20] Conner TS, Brookie KL, Richardson AC, Polak MA (2015). On carrots and curiosity: Eating fruit and vegetables is associated with greater flourishing in daily life. Br. J. Health Psychol..

[21] Głąbska D, Guzek D, Groele B, Gutkowska K (2020). Fruit and vegetable intake and mental health in adults: A systematic review. Nutrients.

[22] Begdache L, Sadeghzadeh S, Derose G, Abrams C (2021). Diet, exercise, lifestyle, and mental distress among young and mature men and women: A repeated cross-sectional study. Nutrients.

[23] Kroes MCW (2014). Food can lift mood by affecting mood-regulating neurocircuits via a serotonergic mechanism. Neuroimage.

[24] Salamone JD, Correa M (2012). The mysterious motivational functions of mesolimbic dopamine. Neuron.

[25] Aquili L (2020). The role of tryptophan and tyrosine in executive function and reward processing. Int. J. Tryptophan Res..

[26] Cools R (2005). Tryptophan depletion disrupts the motivational guidance of goal-directed behavior as a function of trait impulsivity. Neuropsychopharmacology.

[27] Bromberg-Martin ES, Monosov IE (2020). Neural circuitry of information seeking. Curr. Opin. Behav. Sci..

[28] Charpentier CJ, Bromberg-Martin ES, Shea LD (2018). Valuation of knowledge and ignorance in mesolimbic reward circuitry. Proc. Natl. Acad. Sci..

[29] Gruber MJ, Gelman BD, Ranganath C (2014). States of curiosity modulate hippocampus-dependent learning via the dopaminergic circuit. Neuron.

[30] Bordier C, Klein S, Le-Conte Y, Barron AB, Alaux C (2018). Stress decreases pollen foraging performance in honeybees. J. Exp. Biol..

[31] Roberts CJ, Campbell IC, Troop N (2014). Increases in weight during chronic stress are partially associated with a switch in food choice towards increased carbohydrate and saturated fat intake. Eur. Eat. Disord. Rev..

[32] Terenzi D, Liu L, Bellucci G, Park SQ (2021). Determinants and modulators of human social decisions. Neurosci. Biobehav. Rev..

[33] Strang S (2017). Impact of nutrition on social decision making. Proc. Natl. Acad. Sci. USA.

[34] Liu L (2021). Eating to dare: Nutrition impacts human risky decision and related brain function. Neuroimage.

[35] Lydon-Staley DM, Zurn P, Bassett DS (2020). Within-person variability in curiosity during daily life and associations with well-being. J. Pers..

[36] Kobayashi K, Ravaioli S, Baranès A, Woodford M, Gottlieb J (2019). Diverse motives for human curiosity. Nat. Hum. Behav..

[37] van Lieshout, L. L. F., Traast, I. J., de Lange, F. P. & Cools, R. *Curiosity or savouring? Information seeking is modulated by both uncertainty and valence.* https://psyarxiv.com/5y6pz/ (2019). 10.1371/journal.pone.0257011 PMC8462690 34559816

[38] Marvin CB, Shohamy D (2016). Curiosity and reward: Valence predicts choice and information prediction errors enhance learning. J Exp Psychol Gen.

[39] van Lieshout LLF, de Lange FP, Cools R (2021). Uncertainty increases curiosity, but decreases happiness. Sci. Rep..

[40] Morelli SA, Sacchet MD, Zaki J (2015). Common and distinct neural correlates of personal and vicarious reward: A quantitative meta-analysis. Neuroimage.

[41] O’Day EB, Heimberg RG (2021). Social media use, social anxiety, and loneliness: A systematic review. Comput. Hum. Behav. Reports.

[42] Ingram, J., Maciejewski, G. & Hand, C. J. Changes in diet, sleep, and physical activity are associated with differences in negative mood during COVID-19 lockdown. *Front. Psychol.* **0**, 2328 (2020). 10.3389/fpsyg.2020.588604 PMC7492645 32982903

[43] Marty L, de Lauzon-Guillain B, Labesse M, Nicklaus S (2021). Food choice motives and the nutritional quality of diet during the COVID-19 lockdown in France. Appetite.

[44] Jacques A (2019). The impact of sugar consumption on stress driven, emotional and addictive behaviors. Neurosci. Biobehav. Rev..

[45] Hartmann H (2020). Preliminary evidence for an association between intake of high-fat high-sugar diet, variations in peripheral dopamine precursor availability and dopamine-dependent cognition in humans. J. Neuroendocrinol..

[46] Wurtman RJ (2003). Effects of normal meals rich in carbohydrates or proteins on plasma tryptophan and tyrosine ratios. Am. J. Clin. Nutr..

[47] Bromberg-Martin ES, Hikosaka O (2011). Lateral habenula neurons signal errors in the prediction of reward information. Nat. Neurosci..

[48] Vellani V, de Vries LP, Gaule A, Sharot T (2020). A selective effect of dopamine on information-seeking. Elife.

[49] Livermore JJ (2021). Selective effects of serotonin on choices to gather more information. J. Psychopharmacol..

[50] Lang G, Bachinger A (2016). Validation of the German Warwick-Edinburgh Mental Well-Being Scale (WEMWBS) in a community-based sample of adults in Austria: a bi-factor modelling approach. J Public Health.

[51] Döring N, Bortz J (1993). Psychometrische Einsamkeitsforschung: Deutsche Neukonstruktion der UCLA Loneliness Scale [Psychometric research on loneliness: A new German version of the University of California at Los Angeles (UCLA) Loneliness Scale]. Diagnostica.

[52] Hautzinger, M., Bailer, M., Worall, H. & Keller, F. Beck-Depressions-Inventar (BDI). (1994).

[53] Grimm, J. *State-Trait-Anxiety Inventory nach Spielberger. Deutsche Lang- und Kurzversion.* (2009).

[54] Fliege H, Rose M, Arck P, Levenstein S, Klapp BF (2001). Validierung des ‘Perceived Stress Questionnaire’ (PSQ) an einer deutschen Stichprobe. Diagnostica.

[55] Janke S, Glöckner-Rist A (2014). Deutsche Version der Positive and Negative Affect Schedule (PANAS). Zusammenstellung sozialwissenschaftlicher Items und Skalen.

[56] Dehne LI, Klemm C, Henseler G, Bögl KW, Hermann-Kunz E. (1997). Der Bundeslebensmittelschlüssel (BLS II.2). Bundesgesundheitsblatt.

[57] Banna, J. C., McCrory, M. A., Fialkowski, M. K. & Boushey, C. Examining plausibility of self-reported energy intake data: Considerations for method selection. *Front. Nutr.* **4**, (2017). 10.3389/fnut.2017.00045 PMC5622407 28993807

[58] Dixon WJ, Tukey JW (1968). Approximate behavior of the distribution of winsorized t (trimming/winsorization 2). Technometrics.

[59] Willett WC, Howe GR, Kushi LH (1997). Adjustment for total energy intake in epidemiologic studies. Am. J. Clin. Nutr..

[60] Bates D, Mächler M, Bolker B, Walker S (2015). Fitting linear mixed-effects models using lme4. J. Stat. Softw..

[61] Kuznetsova A, Brockhoff PB, Christensen RHB (2017). lmerTest Package: Tests in linear mixed effects models. J. Stat. Softw..

[62] Lenth, R. V., Singmann, H., Love, J., Buerkner, P. & Herve, M. emmeans: Estimated marginal means, aka least-squares means. (2018) 10.1080/00031305.1980.10483031>.License.

[63] Long, J. A. Interactions: Comprehensive, user-friendly toolkit for probing interactions. (2019).

[64] Sjoberg, D. D. *et al.* gtsummary: Presentation-ready data summary and analytic result tables. (2021).

[65] Wickham, H. ggplot2: Elegant graphics for data analysis. (2016).

[66] Revelle, W. psych: Procedures for personality and psychological research. https://CRAN.R-project.org/package=psych (2020).

