# Supplementary material for: Curiosity for information predicts wellbeing mediated by loneliness during COVID-19 pandemic
Source: Sci Rep. 2022 May 11;12:7771. doi: 10.1038/s41598-022-11924-z (PMC9094121; doi:10.1038/s41598-022-11924-z)
Supplement: Supplementary file 1 — Supplementary Information. [file 41598_2022_11924_MOESM1_ESM.pdf]

Supplementary Information

Curiosity for information predicts wellbeing mediated by loneliness during COVID-19 Pandemic

Losecaat Vermeer, A.B. [1, 2 *], Muth, A.[1,2], Terenzi, D. [1,2,3], Park, S.Q. [1,2,3] *

[1] Department of Decision Neuroscience and Nutrition, German Institute of Human Nutrition Potsdam-Rehbrücke, Germany

[2] Charité-Universitätsmedizin Berlin, Corporate member of Freie Universität Berlin, Humboldt-Universität zu Berlin, Berlin, Germany

[3] Deutsches Zentrum für Diabetes, Neuherberg, Germany

Results

Information-seeking motives

Table S1. Mixed effect model for information-seeking

Predictors	Estimates	95% CI	Statistic	p-value
Intercept	66.22	63.91, 68.52	56.70	<0.001
Valence	7.81	5.92, 9.70	8.16	<0.001
Information target	3.17	1.78, 4.57	4.48	<0.001
Valence * Information target	1.11	0.06, 2.14	2.10	0.037
$N_{subjects}$	183			
Marginal R^2 / Conditional R^2	0.114 / 0.677			

Note. Contrasts were set-to-sum. Positive valence and Self as Information target were set to 1.

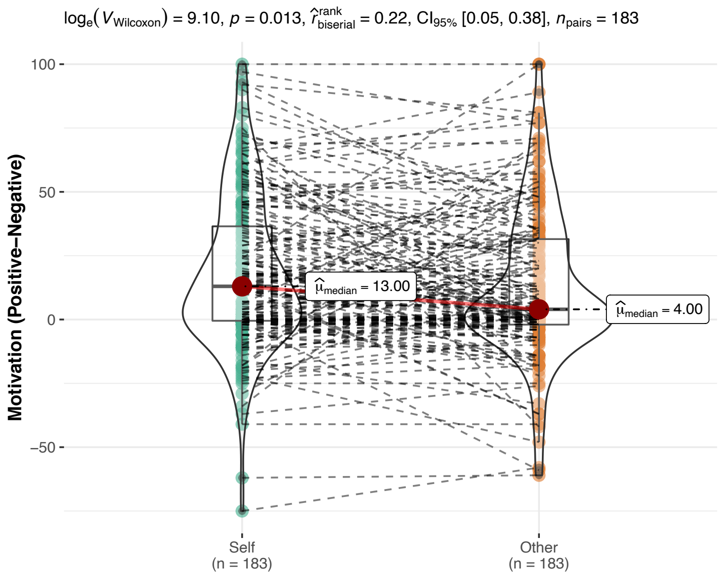

Figure S1. Motivation to seek information that is expected to be positive rather than negative, for Self and about Others.

Curiosity, well-being, and subjective loneliness: preregistered multiple regressions

In the paper, we reported how the link between curiosity and well-being was mediated by loneliness. As loneliness is integral to the pandemic lockdown and was also found to significantly correlate with both the dependent variable well-being and curiosity variables (independent

variables) in the current study, we opted to test for a mediation of loneliness instead of the initially preregistered multiple linear regression with loneliness included as a covariate. For completeness, we report the results of the preregistered regressions below for each curiosity measure. Importantly, the results below overlap with the mediations reported in the paper (i.e. a partial mediation of loneliness with trait curiosity as predictor, and a full mediation of loneliness for information-seeking as predictor of well-being).

Table S2. Multiple regression for well-being (baseline) with trait curiosity as predictor including loneliness, age, gender, and residency as covariates

	Well-being (baseline)			
Predictors	Estimates	95% CI	Statistic	p-value
(Intercept)	44.64	38.54, 50.74	14.44	<0.001
Trait curiosity	4.76	3.45, 6.06	7.18	<0.001
Loneliness (UCLA)	-0.28	-0.34, -0.21	-8.52	<0.001
Age	0.02	-0.09, 0.12	0.31	0.753
Gender	-0.37	-1.32, 0.58	-0.76	0.446
Residency	0.21	-0.79, 1.21	0.42	0.677
Observations	183			
R^2 / R^2 adjusted	0.533 / 0.520			

Table S3. Multiple regression for well-being (baseline) with information-seeking as predictor including loneliness, age, gender, and residency as covariates

	Well-being (baseline)			
Predictors	Estimates	95% CI	Statistic	p-value
(Intercept)	60.70	54.67, 66.72	19.87	<0.001
Information-seeking self	0.02	-0.04, 0.08	0.60	0.552
Loneliness (UCLA)	-0.35	-0.42, -0.28	-10.06	<0.001
Age	0.05	-0.06, 0.17	0.90	0.371
Gender	-0.88	-1.95, 0.18	-1.63	0.105
Residency	0.93	-0.19, 2.05	1.63	0.104
Observations	183			
R^2 / R^2 adjusted	0.399 / 0.382			

Daily well-being and mood

Information-seeking for self on average: including loneliness and covariates for age, gender, residency into the model information-seeking for self did not significantly predict daily mental well-being ($F(1,166) = 0.014$, $p = .907$), daily excitement ($F(1,166) = 1.96$, $p = .163$), and daily anxiety ($F(1,165) = 0.14$, $p = .705$).

Curiosity and daily food intake

Table. S4. Descriptives of food composition and behavior, split for gender

Characteristic	**Overall**, *N* = 172[1]	**male**, *N* = 103[1]	**female**, *N* = 69[1]	**p-value**[2]
Calories	1,659.23 (483.42)	1,787.20 (465.34)	1,467.28 (448.00)	<0.001
Fat (%)	35.48 (6.43)	35.53 (5.45)	35.40 (7.72)	0.9
Carbs (%)	46.34 (7.06)	45.66 (6.79)	47.36 (7.39)	0.12
Protein (%)	16.82 (3.47)	17.61 (3.72)	15.64 (2.68)	<0.001
Sugars (%)	5.92 (3.13)	5.37 (3.08)	6.76 (3.03)	0.004
Fruit & Vegetables (g/1000kcal)	104.18 (92.74)	80.74 (78.27)	139.35 (101.83)	<0.001
Curiosity (trait)	3.08 (0.75)	3.11 (0.65)	3.02 (0.88)	0.4
Information-seeking self	69.25 (18.17)	67.29 (18.31)	72.17 (17.70)	0.085
Daily well-being	22.36 (3.04)	22.49 (3.02)	22.16 (3.09)	0.5
Daily excitement	3.04 (0.65)	3.09 (0.59)	2.98 (0.72)	0.3
Daily anxiety	1.85 (0.64)	1.73 (0.63)	2.02 (0.62)	0.003

[1] Mean (SD), by gender outlier values are winsorized
[2] Two Sample t-test
% daily energy derived

Table S5. Full model predicting daily well-being levels

	**Daily well-being (mean)**			
Predictors	Estimates	95% CI	Statistic	p-value
(Intercept)	27.38	-16.37, 71.14	1.24	0.218
Curiosity (trait)	-1.39	-14.79, 12.01	-0.21	0.838
Carbohydrates	-0.09	-0.62, 0.44	-0.33	0.739
Fat	0.02	-0.57, 0.61	0.07	0.948
Sugar	-0.34	-1.18, 0.50	-0.81	0.421
Fruit and Vegetables	0.01	-0.02, 0.03	0.58	0.560
Age	-0.00	-0.05, 0.05	-0.08	0.933
Gender	0.26	-0.20, 0.73	1.12	0.265
Residency (GER, AT)	-0.03	-0.50, 0.45	-0.11	0.912
Loneliness	-0.08	-0.11, -0.05	-5.7	**<0.001**
Curiosity (trait) * Carbohydrates	0.05	-0.12, 0.21	0.56	0.575
Curiosity (trait) * Fat	-0.00	-0.18, 0.18	-0.03	0.972
Curiosity (trait) * Sugar	0.12	-0.13, 0.38	0.94	0.347
Curiosity (trait) * Fruit and Vegetables	-0.00	-0.01, 0.01	-0.61	0.545
Observations	170			
R^2 / R^2 adjusted	0.345 / 0.290			

Note: Trait curiosity remained significant ($t(164)=3.72$, $p<.001$) in the reduced (winning) model (including loneliness, age, gender, residency) based on model comparison, but for completeness of the food variables, we report the full model here.

Table S6. Full model predicting daily excitement levels

	**Daily excitement (mean)**			
Predictors	Estimates	95% CI	Statistic	p-value
(Intercept)	2.80	-7.45, 13.04	0.54	0.591
Curiosity (trait)	0.50	-2.64, 3.63	0.31	0.755
Carbohydrates	0.00	-0.12, 0.13	0.08	0.940
Fat	0.03	-0.11, 0.17	0.41	0.683
Sugar	-0.11	-0.30, 0.09	-1.10	0.275
Fruit and Vegetables	0.00	-0.01, 0.01	0.11	0.915
Age	-0.02	-0.03, -0.01	-3.06	**0.003**
Gender	0.01	-0.10, 0.12	0.20	0.839
Residency (GER, AT)	0.04	-0.08, 0.15	0.63	0.529
Loneliness	-0.01	-0.02, -0.00	-2.97	**0.003**
Curiosity (trait) * Carbohydrates	-0.00	-0.04, 0.04	-0.13	0.894
Curiosity (trait) * Fat	-0.01	-0.05, 0.03	-0.49	0.627
Curiosity (trait) * Sugar	0.03	-0.03, 0.09	0.87	0.387
Curiosity (trait) * Fruit and Vegetables	0.00	-0.00, 0.00	0.20	0.842
Observations	170			
R^2 / R^2 adjusted	0.211 / 0.146			

Note: Trait curiosity remained significant ($t(164) = 2.41$, $p = 0.017$) in the winning (reduced) model (including loneliness, age, gender, residency) based on model comparison, but for completeness of the food variables, we report the full model here.

Table S7. Full model predicting daily anxiety levels

	**Daily anxiety (mean)**			
Predictors	Estimates	95% CI	Statistic	p-value
(Intercept)	-2.43	-11.96, 7.09	-0.50	0.615
Curiosity (trait)	1.09	-1.82, 4.00	0.74	0.461
Carbohydrates	0.05	-0.07, 0.16	0.80	0.426
Fat	0.00	-0.13, 0.13	0.03	0.980
Sugar	0.25	0.07, 0.43	2.69	**0.008**
Fruit and Vegetables	-0.00	-0.01, 0.00	-1.39	0.167
Age	-0.00	-0.01, 0.01	-0.74	0.459
Gender	-0.11	-0.21, 0.00	-1.97	0.050
Residency (GER, AT)	0.02	-0.09, 0.12	0.29	0.768
STAI (trait)	0.02	0.01, 0.03	3.65	**<0.001**
Loneliness	0.00	-0.00, 0.01	1.10	0.275
Curiosity (trait) * Carbohydrates	-0.02	-0.05, 0.02	-0.87	0.383
Curiosity (trait) * Fat	0.00	-0.04, 0.04	0.05	0.958
Curiosity (trait) * Sugar	-0.08	-0.13, -0.02	-2.71	**0.007**
Curiosity (trait) * Fruit and Vegetables	0.00	-0.00, 0.00	1.09	0.278
Observations	170			
R^2 / R^2 adjusted	0.302 / 0.239			

Methods

Table and plots were created using "gtsummary" [1], "sjPlot" [2] and "ggstatsplot" [3].

Questionnaires

COVID-19 impact

We assessed the impact COVID-19 had on participants' lives by asking them to rate how well they complied with the rules, their current work situation (e.g. home-office), and whether they or others they knew contracted the virus (data presented in Figure S2, Table S8):

- Work situation: "Arbeiten Sie zur Zeit im Homeoffice bzw. arbeiten/studieren/lernen Sie von zu Hause aus?" ["Do you currently work from home, e.g., work/study/learn from home?"] (answers: 1=Yes, 2=No, I go to the office, 3=No, I am not working at the moment, 4=No, I have lost my job due to COVID-19).

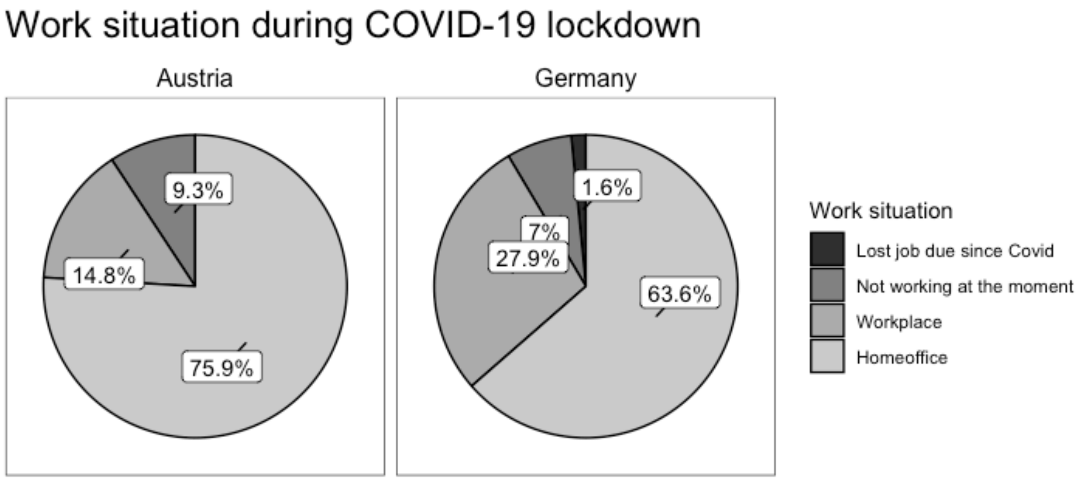

Figure S2. Work situation of the German and Austrian sample. The pie charts illustrate that majority of the participants in our study were working from home.

- Compliance to COVID-19 restrictions, setup by the Government: "Wie streng befolgen Sie die von der Regierung beschlossenen Regeln und Empfehlungen bezüglich der Einschränkung sozialer Kontakte?" ["How strictly do you follow the rules and recommendations adopted by the government regarding the restriction of social contacts?" (answer: 0 = not at all, 100 = very strict"].

- Experience with virus (direct, indirect): (1) "Gibt es in Ihrem näheren persönlichen Umfeld eine an Covid-19 erkrankte Person?" ["Is there someone in your immediate personal environment who has Covid-19?"] (answer options: 1=Yes, in my environment; 2=Yes, I am or was sick with Covid19 myself; 3=No); (2) "Kümmern oder kümmerten Sie sich um eine an Covid-19 erkrankte Person?" ["Do

you take care of a person suffering from Covid-19?”] (answer options: 1=Yes, currently; 2=Yes, in the past; 3=No).

Table S8. COVID-19 impact, split by country

	**Overall**, N = 183[1]	**Austria**, N = 54[1]	**Germany**, N = 129[1]	**P** [2]
Follows Covid-19 restrictions	82.33 (19.71)	84.07 (15.15)	81.60 (21.34)	>0.9
Covid-19 infection - self	2 / 183 (1.1%)	2 / 54 (3.7%)	0 / 129 (0%)	0.086
Covid-19 infection - social network	41 / 183 (22%)	12 / 54 (22%)	29 / 129 (22%)	>0.9
Covid-19 patient caretaker				0.8
- Yes, currently	2 / 183 (1.1%)	1 / 54 (1.9%)	1 / 129 (0.8%)	
- Yes, previously	3 / 183 (1.6%)	1 / 54 (1.9%)	2 / 129 (1.6%)	
- No	178 / 183 (97%)	52 / 54 (96%)	126 / 129 (98%)	

[1] Mean (SD); n / N (%)
[2] Wilcoxon rank sum test; Fisher's exact test; Pearson's Chi-squared test

There were no statistical differences between the Austrian and German samples concerning the impact of COVID-19.

Information-seeking motivation

Four questions assess how willing individuals are to seek information regarding positive and negative information, for themselves and about others in current times. For all questions, participants are asked to rate their motivation using a slider from I am definitely not motivated to look for information (0) to I am highly motivated to look for information (100) [German: Ich bin gar nicht motiviert nach Informationen zu suchen (0) -- Ich bin sehr motiviert nach Informationen zu suchen (100)]. See below.

Wie viele Informationen suchen Sie in AKTUELLEN Zeiten (z.B. im Internet, durch Lesen, Fernsehen, Fragen an Freunde/Familie/etc.). [How much information do you look for in CURRENT times (e.g. on the Internet, by reading, watching TV, asking friends / family / etc.).]

1. Wie sehr sind Sie gewillt, nach Informationen zu suchen, wenn diese Informationen wahrscheinlich <u>POSITIV</u> sind? (also gute Nachrichten, z.B. bezüglich Covid-19, Geld gewinnen; Gehaltserhöhung; guter medizinischer Screening-Test) [How motivated are you to seek information, when that information is likely <u>POSITIVE</u>? (i.e. good news, e.g. regarding Covid-19, gain money; salary increase; good medical screening test)]
2. Wie sehr sind Sie gewillt, nach Informationen zu suchen, wenn diese Informationen wahrscheinlich <u>NEGATIV</u> sind? (also schlechte Nachrichten, z.B. bezüglich Covid-19, Geld verlieren; Lohnkürzung erhalten; schlechter medizinischer Screening-Test) [How motivated

are you to seek information, when that information is likely NEGATIVE? (i.e. bad news, e.g., regarding Covid-19, losing money; receive a wage cut; bad medical screening test)]

3. Wie sehr sind Sie gewillt, nach Informationen zu suchen, wenn diese Informationen über Andere wahrscheinlich POSITIV sind? (also gute Nachrichten) [How motivated are you to seek information, when that information about others is likely POSITIVE? (i.e. good news about others)]
4. Wie sehr sind Sie gewillt, nach Informationen zu suchen, wenn diese Informationen über Andere wahrscheinlich NEGATIV sind? (also schlechte Nachrichten) [How motivated are you to seek information, when that information about others is likely NEGATIVE? (i.e. bad news about others)]

References

1. Sjoberg DD, Whiting K, Curry M, Lavery JA, Larmarange J. (2021). Reproducible summary tables with the gtsummary package. *The R Journal*, **13**:570–80. https://doi.org/10.32614/RJ-2021-053.
2. Lüdecke D (2021). sjPlot: Data Visualization for Statistics in SocialScience. R package version 2.8.8, https://CRAN.R-project.org/package=sjPlot
3. Patil, I. (2021). Visualizations with statistical details: The 'ggstatsplot' approach. *Journal of Open Source Software*, **6**(61), 3167, doi:10.21105/joss.03167
